# Could *Phlebotomus mascittii* play a role as a natural vector for *Leishmania infantum*? New data

**DOI:** 10.1186/s13071-016-1750-8

**Published:** 2016-08-19

**Authors:** Adelheid G. Obwaller, Mehmet Karakus, Wolfgang Poeppl, Seray Töz, Yusuf Özbel, Horst Aspöck, Julia Walochnik

**Affiliations:** 1 Division of Science, Research and Development, Federal Ministry of Defence and Sports, Rossauer Laende 1, 1090 Vienna, Austria; 2 Institute of Specific Prophylaxis and Tropical Medicine, Medical University of Vienna, Kinderspitalgasse 15, 1090 Vienna, Austria; 3 Department of Parasitology, Ege University Faculty of Medicine, 35100 Bornova, İzmir Turkey; 4 Department of Dermatology, Medical University of Vienna, Waehringer Guertel 18-20, 1090 Vienna, Austria; 5 Near East University, Faculty of Science and Letters, Molecular Biology and Genetics, Nicosia, Cyprus

**Keywords:** Phlebotomine sand fly, *Phlebotomus mascittii*, *Leishmania infantum*, Natural infection, Central Europe, Austria

## Abstract

**Background:**

The occurrence of phlebotomine sand flies in Central Europe was questioned until they were recorded for the first time in Germany in 1999, and ten years later also in Austria. The aim of this study was to investigate sand flies collected in Austria for their carrier status of *Leishmania* spp.

**Findings:**

From 2012 to 2013 field studies were conducted in eastern Austria. Altogether, 22 individuals of sand flies were found, all morphologically identified as *Phlebotomus* (*Transphlebotomus*) *mascittii* Grassi, 1908. Twelve non-engorged female specimens with no visible remnants of a blood meal in their bodies were individually investigated for *Leishmania* spp. by ITS-1 real-time PCR. One out of these was positive for *Leishmania*, identified as *Leishmania infantum* by DNA sequencing. This finding suggests that *L. infantum* is not excreted by *P. mascittii* and possibly can establish an infection within *P. mascittii*. Interestingly, an asymptomatic dog living on the farm where this sand fly had been caught was also *Leishmania*-positive.

**Conclusions:**

This study provides new data on the suspected vector capacity of *P. mascittii*, being the northernmost sand fly species in Europe and in most central European regions the only sand fly species found. Proven vector capacity of *P. mascittii* for *Leishmania* spp. would be of significant medico-veterinary importance, not only with respect to expanding sand fly populations in Central Europe related to global warming, but also in the light of globalization and increasing movements of humans.

## Background

Leishmaniasis is a sand fly-borne disease caused by intracellular protozoan parasites of the genus *Leishmania*, with at least 15 species being pathogenic for humans [1]. According to the World Health Organisation, 98 countries, mostly in tropical and subtropical regions, are considered endemic, with 350 million people living at risk of infection and estimated 1.3 million new cases and 20,000–30,000 deaths annually [2]. The clinical spectrum comprises visceral leishmaniasis (VL) and several different forms of cutaneous (CL) and mucocutaneous leishmaniases (MCL), but infections can also remain entirely asymptomatic. *Leishmania* spp. are transmitted by female phlebotomine sand flies (Diptera: Psychodidae: Phlebotominae). Among more than 800 recognized species of sand flies, 78 species are proven vectors of *Leishmania* spp. [3].

The two endemic European transmission cycles include zoonotic VL and CL caused by *L. infantum* throughout all southern countries of Europe and anthroponotic CL caused by *L. tropica* sporadically occurring in Greece. Moreover, *L. donovani*, causing VL and CL, has been recently introduced to Cyprus [4]. In the Mediterranean region, 1200–2000 human cases of VL and 239,500–393,600 cases of CL are estimated to occur annually [5], with a multiple of asymptomatic infections estimated to occur per each symptomatic case [6]. In southern Europe, dogs are the main reservoirs of *L. infantum* [7]. The principal vectors for *L. infantum* in the Mediterranean area are *Phlebotomus* species of the subgenera *Larroussius* (*P. ariasi*, *P. neglectus*, *P. kandelaki*, *P. perfiliewi*, *P. perniciosus* and *P. tobbi*) and *Adlerius* (*P. balcanicus*), and *P.* (*Paraphlebotomus*) *sergenti* and *P.* (*Phlebotomus*) *papatasi* are considered the main vectors of *L. tropica* and *L. major*, respectively, in this region [3, 4].

Since the early reports from Switzerland and France [8–10], the occurrence of phlebotomine sand flies north of the Alps and their possible medical relevance has been a matter of debate. Further proof for autochthonous sand fly populations in Central Europe came with records of *Phlebotomus* (*Transphlebotomus*) *mascittii* from Belgium, France, Germany and later also Austria [7, 11–13]. *P. mascittii* was first described by Grassi in 1908 from Rome (Italy), and generally appears to be widespread in Europe, albeit in low population densities and with low rates of biting humans [7]. The vector competence of *P. mascittii* has not been conclusively clarified so far, but *P. mascittii* has been described as a generally aggressive species, has been reported to suck blood on humans [10] and a few assumedly autochthonous cases of leishmaniasis in human and animals have been described in regions where *P. mascittii* is the only sand fly species found, including two cases in Austria (reviewed in [14–16]). Moreover, one female specimen of *P. mascittii* was found positive for *Leishmania* spp. by polymerase chain reaction (PCR) on the island of Montecristo, Italy [17].

In Austria, *P. mascittii* was first recorded in 2009 [12]. Since then, several field studies in various regions of Austria have been conducted [16, 18], and interestingly, *P. mascittii* was only found close to human dwellings, usually farms with livestock and companion animals, corroborating its adaption to man-made environments. The aim of the present study was to investigate Austrian specimens of *P. mascittii* for their carrier status for *Leishmania* spp.

## Methods

### Study area and sand fly sampling

**Fig. 1:**
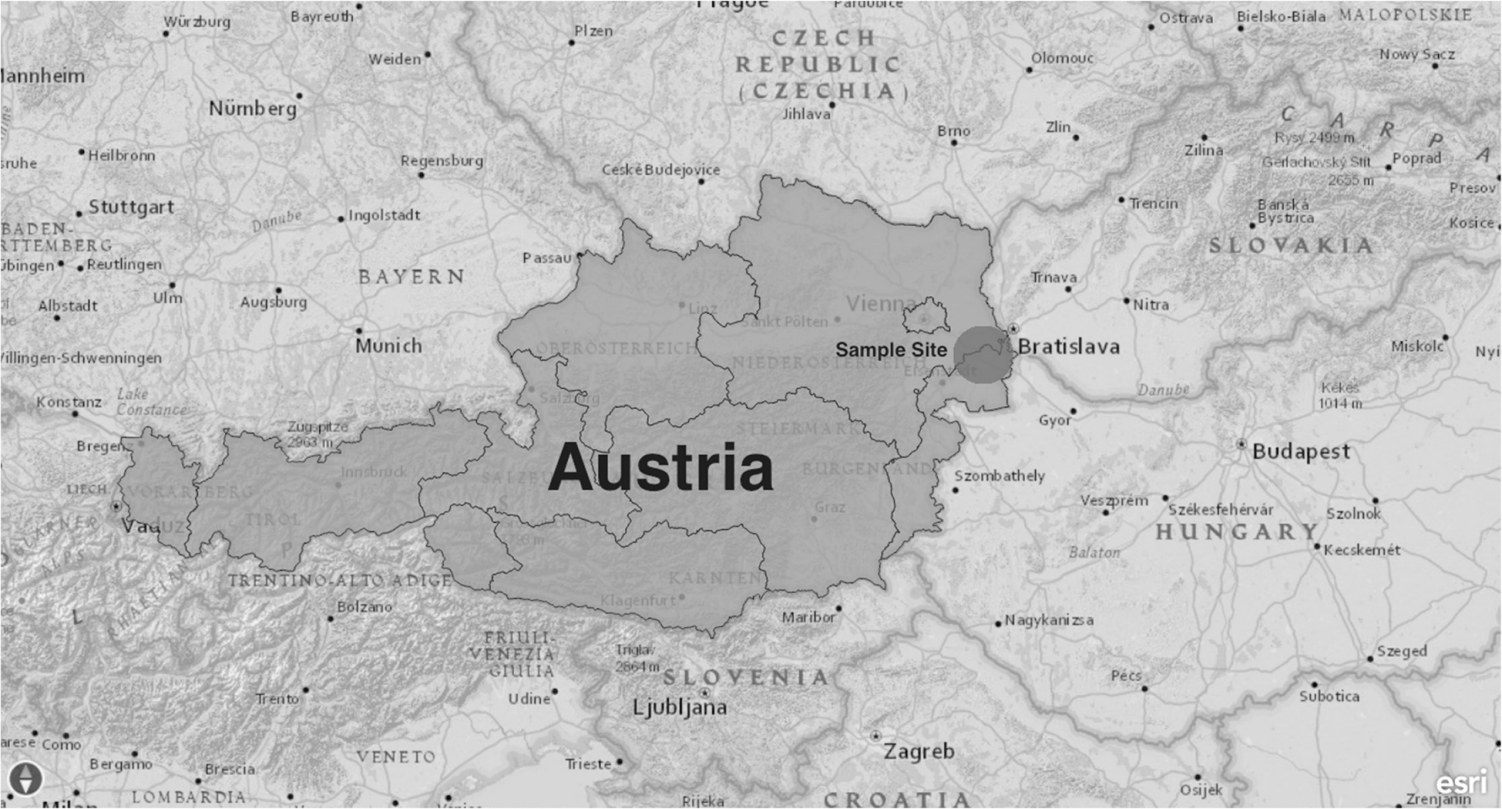
Map showing geographical location of the study area

The sampling site was located in eastern Austria, in the federal state of Lower Austria, around 16 km (linear distance) from the border with Slovakia and 21 km from the border with Hungary (Fig. 1). The area is characterised by a Pannonian climate with continental influence, with hot summers and cold winters. The entomological field studies were performed in the village of Rohrau (48°3′57″N, 16°51′33″E; altitude 148 m) in July and August 2012 and 2013 using 440 light traps in total. At this site, sand flies had already been found before.

Sand flies were trapped and identified as described previously [16, 18]. In brief, we used battery-operated CDC Miniature Light Traps, with an ultra-fine mesh (John W. Hock Company, Gainesville, FL, USA). Sand flies were collected with up to eight traps distributed at the sampling site, a farmhouse with a nineteenth century partially open wooden barn with loamy ground and an open garage with concrete pavement. Horses, several cats and a dog were held on the farm. The trapped sand flies were transferred into 70 % ethanol for further investigation. For identification, specimens were cleared with potassium hydroxide solution and slide-mounted in Hoyer’s fluid. Species identification was based on basic taxonomical structures, mainly, pharynx, male genitalia and female spermathecae using the identification keys by Theodor and Seccombe et al. [19, 20].

### DNA extraction and PCR assays

Males and fed females were excluded from the analysis. All non-engorged female specimens of *P. mascittii* were prepared as follows: thorax and abdomen of the sand flies were individually transferred to ZR Bashing Bead™ tubes (Zymo Research Corporation, Freiburg, Germany) and homogenised using a MagNa Lyser® (7000 rpm 90 s, Roche Molecular Diagnostics, Mannheim, Germany). Male sand fly specimens, a blood sample from a healthy dog and high pure PCR water were used as negative controls. Tubes were incubated overnight at 56 °C after adding 500 μl volume of Qiagen® Tissue lysis buffer (Qiagen Inc., Vienna, Austria). DNA extractions were made using the Qiagen® Tissue&Blood extraction kit with a slight modification on the final elution step (elution in a volume of 50 μl) to obtain high yield of DNA.

Real-time PCR targeting the internal transcribed spacer 1 (ITS-1) region between the 18S and the 5.8S rRNA genes was performed using species-specific primers for detecting and distinguishing *L. donovani/infantum* complex, *L. tropica* and *L. major* [21]. PCR was performed using 20 ng genomic DNA, 10 pmol of each primer, 200 nM of each probe and 20 μl QuantiTect® Probe PCR mix per reaction. Melting curves were generated using channel 2 and 3. Three international reference strains *L. infantum* (MHOM/TN/80/IPT1), *L. tropica* (MHOM/SU/74/SAF-K27) and *L. major* (MHOM/SU/73/5ASKH) were included to obtain standard curves.

A conventional PCR, also targeting the ITS-1 region and using the LITSR/L5.8S primers [22], was performed with a ready-to-use PCR master mix (Helixamp®T500N) to obtain sequence data for the positive sample. PCR products were visualized in a 1.5 % agarose gel using a UView™ Mini Transilluminator. The amplicon was purified using a PCR band extraction kit (QIAquick®, Qiagen) and sequenced commercially. Sequence data were analysed using Geneious R8 [23].

At the time the sand flies were caught, also an EDTA whole blood sample of the dog living at the collection site was taken and also tested for *Leishmania* spp. by conventional ITS-1 PCR as described above. The DNA was extracted from the blood using the peqGOLD Blood DNA Mini Kit (Peqlab, Erlangen, Germany). After PCR, the amplicon was purified and sequenced in both directions using the BigDye® Terminator v1.1 Cycle Sequencing Kit and an automatic 310 ABI PRISM sequencer (Applied Biosystems, Darmstadt, Germany).

As, according to the owners, the approximately one-year-old male dog had never been outside of Austria, EDTA whole blood samples were also obtained from a sibling and the mother bitch, both living in a distant region of Austria and never having been to the farm. These samples were also tested by *Leishmania-*specific PCR as described above. Unfortunately, the samples from the mother bitch and the sibling were lost for follow-up by DNA sequencing. All sequence data obtained were submitted to the GenBank database under accession numbers KT026221 (sand fly) and KU555887 (dog).

## Results and discussion

**Fig. 2:**
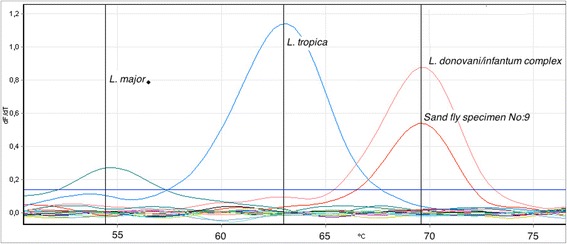
Melting temperature analysis showing the specific melting peak for the *L. donovani/infantum* complex (negative specimens including controls are under the threshold in the graph)

A total of 22 sand flies were trapped during the study period, namely six females and one male in 2012 and 13 females and two males in 2013. All specimens were identified as *Phlebotomus* (*Transphlebotomus*) *mascittii,* the only species known to occur in Austria so far*.* Altogether, 12 female specimens were un-engorged with no visible remnants of a blood meal in their bodies. Out of these, one specimen (specimen No. 9) showed a *L. donovani/infantum* complex-specific melting peak in the ITS-1 real time PCR, with high analogy to the positive control for *L. infantum* (MHOM/TN/80/IPT1) (Fig. 2). The three negative controls and the remaining specimens did not reveal any peaks. As this real time PCR cannot identify below the level of *L. donovani/infantum* complex, we conducted a conventional ITS-1 PCR to obtain sequence data. The obtained sequence from the sand fly showed a 100 % similarity (315/315 bp) to numerous strains of *L. infantum* (e.g. KC477100), including also the strain obtained from the dog living on the farm where the *Leishmania*-positive sand fly had been caught. According to the owners, the approximately 1-year-old male dog had never been outside of Austria. He had lived one transmission season on the farm, spending most of the nights in the open and having access to the barn where the infected sand fly was caught. Our working hypothesis is that the sand fly took up the parasites during a blood meal on the *L. infantum*-positive dog and that the dog had acquired its infection by vertical transmission from the bitch living in a distant farm in Austria. The mother bitch and one of the siblings were also positive for *Leishmania* spp. by PCR and an independent infection seems implausible. The possibility of vertical transmission in canine leishmaniasis has been reported previously [24, 25]. The bitch was used for breeding and had travelled abroad frequently. However, how exactly the bitch became infected cannot be deduced unequivocally; veneral transmission should also be taken into account [25].

The presence of *Leishmania* DNA in *P. mascittii* was first reported from the Island of Montecristo, Italy, in 2014, albeit without details on the feeding status of the respective sand fly [17]. The current study, demonstrating *Leishmania* DNA in an un-engorged specimen of *P. mascittii*, now provides further data on the possible vector capacity of *P. mascittii*, the northernmost sand fly species in Europe, and in most central European regions the only sand fly species found. The established criteria for the incrimination of natural vectors are: (i) anthropophilic behaviour; (ii) repeated isolation of the same species of *Leishmania* from the sand fly as that found in patients; (iii) feeding on reservoir host(s); (iv) development of the parasite in the sand fly; and (v) ability to transmit the parasite by bite to a susceptible host while taking a blood meal [26]. For *P. mascittii*, as outlined in the introduction, the first three of these criteria essentially apply. Our data now suggest that *L. infantum* is at least not excreted with the faeces by *P. mascittii* and might thus be able to establish a long-term infection in *P. mascittii*.

Indicative data for this assumption firstly is that the *Leishmania*-positive sand fly was a clearly un-engorged specimen. In sand flies held at 28 °C, the blood meal is typically digested within 72 h, having a bright red colour during the first 18 h, a dark red colour during 18–24 h and a brown colour between 24 and 72 h [27]. At lower temperatures, defecation is delayed. In a study, in which sand flies were held at 26 °C, the majority of sand flies had lost their blood by days 7–8, only 14–27 % of them still showing remnants of a blood meal [28]. Lower temperatures however, even as low as 20 °C, do not prohibit but only delay *Leishmania* development in sand flies [29]. In the current study, no remnants of a blood meal were visible, thus the blood meal must have occurred at least 72 h, but because of day temperatures around 24 °C and night temperatures around 19 °C [18], rather ≥ 8 days before the trapping. This suggests that the parasites might indeed have established an infection in the sand fly, because otherwise, the parasites would have been defecated with the blood remnants and no DNA would have been detectable. DNA of non-living cells is known to be rapidly degraded in the sand fly gut, e.g. at 28 °C the DNA of cells of the blood host can only be detected until 24 h after engorgement, already after 37 h no host cell DNA is detectable any more [27]. This rapid degradation of DNA is explained by a peak activity of proteases and DNAses between 24 and 48 h after feeding in the sand fly gut [27], which however of course, is also temperature-dependent. Moreover, it is unknown whether the DNA in dying or dead *Leishmania* cells behaves similarly to DNA in dying or dead host cells and amastigote *Leishmania* DNA is not only protected by the *Leishmania* cell but also by the host cell and might thus be detectable for longer periods. In a competent vector, the development of *Leishmania* spp. proceeds in the digestive tract, the precise location differing between species of the subgenera *Leishmania* and *Viannia. Leishmania infantum* is a suprapylarian parasite, developing in the midgut of the sand fly. Surviving the early phase after uptake by the sand fly is the first hurdle parasites must overcome [30]. Many *Leishmania* species are very specifically linked to a certain sand fly species, e.g. *L. tropica* is specifically adapted to *P. sergenti* and *P. sergenti* has been shown to be refractory to *L. major* and *L. donovani* because these two species cannot specifically bind to the midgut epithelium of *P. sergenti* and are thus defecated [28]. The latter study also demonstrated that the loss of infection with *L. major* and *L. donovani* in *P. sergenti* correlates with the excretion of the digested blood meal, which in that study was completed at approximately day 8 after the blood meal. According to Pimenta et al. [31], up to 50 % of the parasites are killed by secreted proteolytic enzymes and excreted during the first day after the blood meal, even in proven vectors. The parasites must then, to establish an infection in the sand fly, attach to the midgut epithelia via specific lipophosphoglycans, constituting an assumed determinant of parasite-vector specificity [28, 30]. In *Lutzomyia migonei*, which has recently been recognized as a permissive vector for *L. infantum*, metacyclic promastigotes develop within five days after the blood meal [32]. In *P. perniciosus*, *L. infantum* starts to proliferate two days after the blood meal and develops heavy late-stage infections within 8 days even at only 20 °C ambient temperature [29]. The missing proof of metacyclic promastigotes from the infected sand fly is the major limitation of the current study. However, the isolation and culture of *Leishmania* spp*.* from dissected wild-caught sand flies is difficult, the prevalence even in proven vectors sometimes is low and thus large numbers of individuals are required. In an Italian study, only one out of 70 dissected sand flies revealed microscopically detectable promastigotes, but 47 % of the sand flies investigated by nested PCR were positive for *Leishmania* spp. [33]. In Austria, the sand fly populations are scattered and extremely small. In the current study we only had 12 un-engorged and seven engorged female specimens available and the entire thorax and abdomen of the sand flies were used for DNA isolation to be as sensitive as possible, as is common practice.

A second valid argument for the possible ability of *L. infantum* to establish within *P. mascittii* is, that *L. infantum* generally seems to have a particular predisposition to be transmitted by multiple vectors. It is the only *Leishmania* species known that can be transmitted by sand flies of the genus *Phlebotomus* as well as by sand flies of the genus *Lutzomyia*. Recently, it has been shown, that not only *Lutzomyia longipalpis* but also *L. migonei* is a permissive vector for *L. infantum* [31]. Thus, it seems conceivable, that this species can also accept various species within the genus *Phlebotomus* as vectors. In southern European countries, proven vectors of *L. infantum* are *P. ariasi*, *P. perniciosus* and *P. neglectus*, but up to 13 more species are considered suspected vectors [1, 26].

Altogether, if *P. mascittii* should really prove to be a permissive vector for *L. infantum*, this would be of significant medical relevance for central Europe, as parasite reservoirs are already given in most regions. In Austria, a study by Poeppl et al. [34] revealed an unexpected high (4.5 %) seropositivity against *Leishmania* spp. in 1048 asymptomatic Austrians, most, of course, with travel history, but two being also PCR-positive, thus potential resevoirs. More importantly, increasing numbers of dogs in Central Europe are infected with *Leishmania* spp., firstly because travelling with animals has become easier since the opening of borders, but secondly also because dogs from endemic areas are sold over the internet and stray dogs are relocated from endemic areas by animal lovers. In 2008, Leschnik et al. [35] examined 119 Austrian symptomatic dogs that had been abroad, 55 of them being positive for *Leishmania* spp. Germany is estimated to have several thousands of infected dogs, the majority coming from Italy, Spain and Portugal [25]. To date, the population sizes of sand flies in Central Europe, including Austria, are still extremely small, but increases in population densities can be expected. In the exceptionally hot summer of 2015 we collected almost as many sand flies in two trapping nights, as we had collected the years before in over 700 trapping nights (unpublished data).

## Conclusions

This study provides new data on the possible vector role of *P. mascittii* for *L. infantum. Phlebotomus mascittii* is the northernmost sand fly species in Europe and in most central European regions the only species found. With currently growing *Leishmania* reservoirs in previously non-endemic regions due to travelling, import and transport of dogs and large-scale movements of people, the evidence for vector capacity of *P. mascittii* would be of high medical importance, particularly as sand fly populations in Central Europe can be expected to expand with global warming.

## Abbreviations

CL: Cutaneous leishmaniasis
ITS-1: Internal transcribed spacer 1
MCL: Mucocutaneous leishmaniasis
PCR: Polymerase chain reaction
VL: Visceral leishmaniasis
WHO: World Health Organisation
